# Recurrent CDK1 overexpression in laryngeal squamous cell carcinoma

**DOI:** 10.1007/s13277-016-4991-4

**Published:** 2016-02-24

**Authors:** K. Bednarek, K. Kiwerska, M. Szaumkessel, M. Bodnar, M. Kostrzewska-Poczekaj, A. Marszalek, J. Janiszewska, A. Bartochowska, J. Jackowska, M. Wierzbicka, R. Grenman, K. Szyfter, M. Giefing, M. Jarmuz-Szymczak

**Affiliations:** 1 Department of Cancer Genetics, Institute of Human Genetics, PAS, Poznan, Poland; 2 Department of Clinical Pathomorphology, Collegium Medicum, Nicolaus Copernicus University, Bydgoszcz, Poland; 3 Department of Oncologic Pathology, Greater Poland Cancer Centre, Poznan, Poland; 4 Department of Otolaryngology and Laryngological Oncology, University of Medical Sciences, Poznan, Poland; 5 Department of Otorhinolaryngology—Head and Neck Surgery and Department of Medical Biochemistry, Turku University Hospital and University of Turku, Turku, Finland; 6 Department of Audiology and Phoniatry, University of Medical Sciences, Poznan, Poland; 7 Department of Hematology, University of Medical Sciences, Poznan, Poland

**Keywords:** *CDK1*, Overexpression, Gene methylation, microRNA, Western blot, Immunohistochemistry

## Abstract

In this study, we analyzed the expression profile of four genes (*CCNA2*, *CCNB1*, *CCNB2*, and *CDK1*) in laryngeal squamous cell carcinoma (LSCC) cell lines and tumor samples. With the application of microarray platform, we have shown the overexpression of these genes in all analyzed LSCC samples in comparison to non-cancer controls from head and neck region. We have selected *CDK1* for further analysis, due to its leading role in cell cycle regulation. It is a member of the Ser/Thr protein kinase family of proven oncogenic properties. The results obtained for *CDK1* were further confirmed with the application of reverse transcription quantitative polymerase chain reaction (RT-qPCR) technique, Western blot, and immunohistochemistry (IHC). The observed upregulation of *CDK1* in laryngeal squamous cell carcinoma has encouraged us to analyze for genetic mechanisms that can be responsible this phenomenon. Therefore, with the application of array-CGH, sequencing analysis and two methods for epigenetic regulation analysis (DNA methylation and miRNA expression), we tried to identify such potential mechanisms. Our attempts to identify the molecular mechanisms responsible for observed changes failed as we did not observe significant alterations neither in the DNA sequence nor in the gene copy number that could underline *CDK1* upregulation. Similarly, the pyrosequencing and miRNA expression analyses did not reveal any differences in methylation level and miRNA expression, respectively; thus, these mechanisms probably do not contribute to elevation of *CDK1* expression in LSCC. However, our results suggest that alteration of *CDK1* expression on both mRNA and protein level probably appears on the very early step of carcinogenesis.

**Electronic supplementary material:**

The online version of this article (doi:10.1007/s13277-016-4991-4) contains supplementary material, which is available to authorized users.

## Introduction

Larynx squamous cell carcinoma (LSCC) is one of the most frequent types of head and neck cancer [1]. The main etiological causes of this type of cancer are well known and include the synergistic effects of tobacco smoking and alcohol abuse [2].

Chromosomal aberrations resulting in alterations in gene expression (downregulation of tumor suppressor genes or overexpression of oncogenes) are one of the genetic factors contributing to the development of this cancer. Until now, several oncogenes have been shown to be involved in the development of head and neck cancers including *CCND1*, *FGF3*, *FGF4*, *CTTN* [3], or *EGFR* [4]. In our previous work, we have shown also other oncogenes like the *ORAOV1*, *FADD* [5], and *CRKL* [6] as potentially related to LSCC.

As aberrant cell cycle control plays a significant role in tumor development, we focused on the genes involved in this proces. We analyzed the expression level of group of four genes (*CDK1*, *CCNB1*, *CCNB2*, and *CCNA2*) engaged in cell cycle control. *CDK1* (cyclin-dependent kinase 1) gene is an important factor of the cell cycle control system [7, 8]. Together with cyclin B (*CCNB1* and *CCNB2*), it forms an active MPF (maturation-promoting factor) which makes cell enter mitosis [9]. The A cyclin (CCNA2) binds CDK1 during the transition from G2 to M phase [10]. Moreover, *CDK1* is required for mammalian cell proliferation as it is the only CDK that can initiate the onset of mitosis [11]. For all these genes, we observed gene upregulation in LSCC cell lines and tumor samples as compared to non-cancer controls. However, due to the leading role of *CDK1* in cell cycle control and regulation, we have chosen the *CDK1* for further analysis. This gene is a member of the Ser/Thr protein kinases family. The oncogenic potential of these enzymes was demonstrated [12–14]. The CDK1 protein is a catalytic subunit of M-phase promoting factor essential for the G1/S and G2/M transition in eukaryotic cell cycle.

Interesingly, in model organism *Saccharomyces cerevisiae*, the 75 target genes of Cdk1 were shown to control cell cycle, DNA replication and segregation, transcriptional programs, and cell morphogenesis [15]. These findings suggest that deregulation in *CDK1* gene may play a significant role in cancerogenesis. Moreover, the involvement of *CDK1* in tumorgenesis was postulated in various types of cancer, including laryngeal cancer [16, 17].

Due to the suggested role of *CDK1* in tumorigenesis, we analyze here the *CDK1* DNA copy number, gene sequence, DNA methylation status, and miRNA expression profile with the aim to identify the responsible mechanism for the observed *CDK1* upregulation in LSCC.

## Materials and methods

### Cell lines

Twenty-five cell lines derived from laryngeal cancer were used. The cell line characteristics are shown in Table 1.

**Table 1:** Cell line characteristics

Cell line number	Sex	Age (years)	Primary tumor location	TNM	Specimen site	Type of lesion	Grade	Survival
UT-SCC-6A	F	51	Supraglottic larynx	T_2_N_1_M_0_	Larynx	rec	G1	DWD 31 months
UT-SCC-6B	F	51	Supraglottic larynx	T_2_N_1_M_0_	Neck	met	G1	DWD 31 months
UT-SCC-8	M	42	Supraglottic larynx	T_2_N_0_M_0_	Larynx	pri	G1	DWD 35 months
UT-SCC-11	M	58	Glottic larynx	T_1_N_0_M_0_	Larynx	rec	G2	DNE >5 years
UT-SCC-13	M	53	Supraglottic larynx	T_3_N_0_M_0_	Larynx	rec	G2	DWD 11 months
UT-SCC-19A	M	44	Glottic larynx	T_4_N_0_M_0_	Larynx	pri	G2	DNE >5 years
UT-SCC-19B	M	44	Glottic larynx	T_4_N_0_M_0_	Larynx	pri (per)	G2	DNE >5 years
UT-SCC-22	M	79	Glottic larynx	T_1_N_0_M_0_	Larynx	rec	G2	DWD 28 months
UT-SCC-23	M	66	SCC transglottic	T_3_N_0_M_0_	Larynx	pri (per)	G1	DNE >5 years
UT-SCC-29	M	82	Glottic larynx	T_2_N_0_M_0_	Larynx	pri	G1	DNE 10 years and 4 months
UT-SCC-34	M	63	Supraglottic larynx	T_4_N_0_M_0_	Supraglottic larynx	pri	G1	DWD 10 months
UT-SCC-35	M	50	Glottic larynx	T_2_N_0_M_0_	Larynx	resid	G2	DWD 10 months
UT-SCC-38	M	66	Glottic larynx	T_2_N_0_M_0_	Larynx	pri	G2	DWD 16 months
UT-SCC-42B	M	43	Supraglottic larynx	T_4_N_3_M_0_	Neck	pri	G3	DWD 2 months
UT-SCC-49	M	76	Glottic larynx	T_2_N_0_M_0_	Larynx	pri	G2	DWD 2 years and 7 months
UT-SCC-50	M	70	Glottic larynx	T_2_N_0_;rT_2_N_0_	Larynx	rec	G3	ANE >5 years
UT-SCC-57	M	76	Glottic larynx	T_2_N_0_M_0_	Larynx	rec	G1-G2	DWD 4 years
UT-SCC-75	M	56	SCC laryngis	T_2_N_2B_M_0_	Larynx	pri	G2	D, NED 2 years and 6 months
UT-SCC-106A	M	59	SCC plicae vocalis	T_1A_N_0_M_0_	Larynx	pri	G2	DNE second pri 4 years and 1 month
UT-SCC-106B	M	59	SCC plicae vocalis	rT_3_N_0_M_0_	Larynx	rec	G3	DWD 5 days
UT-SCC-107	M	46	SCC laryngis supraglottis	T_4_N_2C_M_0_	larynx	pri	G2	DNE 19 months
UT-SCC-108	M	68	SCC laryngis supraglottis	T_2_N_0_M_0_	larynx	pri	G3	DWD 19 months
UT-SCC-113	M	50	SCC laryngis transglottica	T_3_N_0_M_0_	larynx	pri	G3	DWD 17 months
UT-SCC-116	M	60	SCC laryngis supraglottis	T_4_N_1_M_0_	larynx	pri	G2	DWD 9 months
UT-SCC-117	M	71	SCC laryngis (resid T_2_N_0_M_0_)	T_2_N_0_M_0_	Larynx	rec	G2	DWD 47 months

All cell lines were obtained in University of Turku (Finland)

*M* male, *F* female, *TNM* TNM classification (*T* tumor, *N* lymph node involvement, *M* distance metastases), *pri* primary tumor, *rec* recurrence, *met* metastasis, *per* persistent tumor, *DWD* died with the disease, *DNE* died with no disease evident, *ANE* alive with no disease evident

## Primary tumor samples

### Primary tumor samples used for the mRNA and microRNA expression analysis and pyrosequencing

Forty-five laryngeal cancer samples (1 female and 44 males) were used in the study. The average patients age was 61 years (ranged 42–84). The TNM and G status details are shown in Table S1. During the surgery, each sample was divided into three parts and designated for histopathological analysis, DNA isolation (immediate freezing in −80 °C), and RNA analysis (storage in RNAlater, Sigma, according to manufacturer’s instruction). Only samples containing more than 60 % of tumor cells were chosen for the study. The study was approved by the local ethical board of Medical University in Poznan. Written consent was obtained from all donors.

### Primary tumor samples used for immunohistochemistry

The studies were performed on a group of 40 patients (5 females, age, 50–69 and 35 males, age, 44–77), who underwent total laryngectomy. Based on histopathological examination, performed by two independent pathologists, in all cases, laryngeal squamous cell carcinoma (LSCC) was diagnosed. The tumor stage was determined according to the current TNM classification published by the International Union Against Cancer (IUAC). The TNM and G status details are shown in Table S1.

The immunohistochemical studies were performed on selected archival formalin-fixed paraffin embedded (FFPE) tissue sections. All cases were revised and selected by two independent pathologists according to hematoxylin and eosin-stained tissue sections. In each sample, cancer cells occupied approximately 80 % of tissue area.

## Control samples

### Control samples used for the expression analysis

Various types of non-malignant samples were used as controls for microarray and reverse transcription quantitative polymerase chain reaction (RT-qPCR) expression analysis: the commercially available human total RNA derived from healthy larynx (Total Larynx RNA, Stratagene, Agilent Technologies, Waldbronn, Germany), the RNA derived from bronchial airway epithelia reconstituted in vitro (two donors) (EC, Epithelix Sarl, Geneve, Switzerland), normal mucosa derived from surgical margin during laryngectomy (LX10), normal human bronchial/tracheal epithelial cells—NHBE (Lonza, Verviers, Belgium), and human tracheal epithelial cells—HTEC (PromoCell, Heidelberg, Germany).

### Control samples used for *CDK1* gene promoter DNA methylation analysis

Two groups of controls included 20 DNA samples isolated from head and neck region. Ten were derived from the oral cavity epithelium (buccal swabs, W1–W10) from healthy donors. The second group of controls (K1–K10) were collected during surgeries not associated with cancer and include samples from the resected epithelium of Reinke’s edema and normal vocal fold fragments removed during surgical widening of the glottis in patients with bilateral vocal cord paralysis. Additionally, the fully methylated standard (MK, Millipore, Hilden, Germany) and unmethylated DNA (UM), i.e., the whole genome amplified DNA from peripheral blood lymphocytes (prepared with GenomePlex® Whole Genome Amplification Kit) were used in each run.

### Protein lysates used for Western blot analysis

The presence of the examined protein (CDK1) was analyzed in comparison to commercially available total larynx tissue lysates (Larynx Human Tissue Lysates from adult normal tissue—ab44731 and ab44733, pooled, Abcam, UK), four protein lysates obtained from the non-cancer tissue samples (K12-K15), and three lysates from the epithelial cells (primary culture obtained in our laboratory) derived from the non-cancer tissue samples (K2, K3, K6). As positive controls, the Jurkat cell line lysate (Jurkat E6.1, Human T-cell lymphocytes, ECACC, Salisbury, UK) or HeLa lysate (cervical cancer cell line) was used.

### Control samples used for the immunohistochemical analysis of CDK1 protein

The control material for the immunohistochemical studies consisted of archival FFPE tissue sections, revised, and selected by two independent pathologists. The sections contained a disease-free normal mucosa, at least 2 cm distant from the tumor margins. To establish the immunohistochemical protocol, a series of positive and negative control reactions were performed. The positive control reaction was performed on placenta, where the presence of CDK1 protein expression was indicated (reference sources: [18]; the Human Protein Atlas; and manufacturer antibodies datasheet). The negative control reaction was performed by substituting the primary antibody by the 1 % BSA (bovine serum albumine) solution in PBS (phosphate buffered saline).

For all the control samples used in our study, an appropriate agreement from the bioethical board of the Poznan Medical University was obtained.

### Cell culture

The culture condition for cell lines and primary cultures of epithelial cells were described elsewhere with minor modification [5, 19, 20]. For DNA, RNA, and protein isolation, cell lines were cultured to 80 % confluence and then harvested with 0.1 % trypsin and 0.2 % EDTA. The cell lines used as controls for Western blot were cultured under identical conditions, using the modified Eagle medium (MEM) for HeLa and RPMI-1640 medium for the Jurkat cell line, with 100 U/mL penicillin/streptomycin medium, 37 °C, 5 % CO_2_).

### DNA, RNA, and protein isolation

Nucleic acids from cell lines, tumor samples, and peripheral blood lymphocytes were isolated according to standard methods: phenol/chloroform extraction and ethanol precipitation for DNA and Chomczynski’s method with application of Trisol for RNA [21]. Nucleid acids purity and concentration was analyzed as described elsewhere [22].

Total protein extraction from the LSCC cell lines and controls (except tissue samples) was performed with Nonidet-P40 (NP-40) buffer with the Protease Inhibitor Cocktail (LabEmpire, Rzeszow, Poland). Total protein lysates from the non-cancer tissue samples were obtained with the use of FASTPREP-24™ 5G Instrument (MP Biomedicals) after the grinding in liquid nitrogen Protein concentration was measured with standard Bradford method [23].

### Microarray-based gene expression analysis

Expression analysis was performed on GeneChip Human Genome U133 Plus 2.0 Array (Affymetrix) for 10 cell lines (UT-SCC-6A, −11, −19B, −22, −29, −34, −57, −106A, −107, −116), 5 tumor samples (out of 45 collected), and 3 control samples (Total Larynx RNA, EC, LX10). The microarrays were performed and analyzed as described elsewhere [5, 24, 25]. The number of tags used to analyze the expression of chosen genes was as follows: two tags for the *CCNA2* gene (203418_at and 213226_at), one for *CCNB1* (214710_s_at), and one for *CCNB2* (202705_at). Two tags were used to analyze *CDK1* expression: 203213_at and 203214_x_at as they correspond to entire coding exons of the gene. The gene upregulation was defined as increased gene expression observed for analyzed tag in comparison to expression level of this tag in at least two of the three non-cancer controls.

### Reverse transcription and quantitative real-time PCR

Total RNA from 25 LSCC cell lines and 3 non-tumor control samples (Total Larynx RNA, NHBE cells and HTEC cells) were used to synthesize the cDNA template using of Enhanced Avian RT First Strand Synthesis Kit (Sigma-Aldrich), according to manufacturer’s protocol. RT-qPCR primers were designed with the use of Beacon Designer™ 7.5 (PRIMER Biosoft International) and the specificity was verified with the primer BLAST software (http://blast.ncbi.nlm.nih.gov/Blast.cgi). Three genes were used as reference genes: *ARNT*, *UBC*, and *GAPDH*. The primer sequences are listed in Table S2. Quantitative real-time PCR was performed with the iCycler iQ5 (Bio-Rad), and the gene expression profile was analyzed with a detection system using iQ5 Optical System Software 2.0 (Bio-Rad Laboratories). The annealing temperature applied for *CDK1* and reference genes was 55 °C; detailed PCR conditions are presented in Supplementary data. For PCR, the 5× HOT FIREPol® EvaGreen® qPCR Supermix (Solis BioDyne, Estonia) according to the manufacturer’s protocol was used. For each sample, 0.4 μL of cDNA was used (undiluted reverse transcription product derived from 8 μg RNA in 40 μl reaction). The melting curve, determination of PCR efficiency, PCR data analysis, and statistics were previously described [6]. To estimate the changes in gene expression, the cutoff point value was calculated according to the scheme: three-times standard deviation of the controls plus the expression value of the highest control.

### CDK1 protein western blot analysis

The whole cell lysates from 25 cell lines and 8 samples from the non-cancer larynx tissue were used. In each blot, positive control (Jurkat and/or HeLa cell line lysate) was used. Detailed information can be found in the Supplementary data. The rabbit polyclonal anti-CDK1-C-terminal antibody (ab7953, Abcam, UK, dilution 1:1000) was applied for all 25 cell lines and 8 non-cancer larynx tissue lysates. The second, N-terminal antibody (ab131011, Abcam, UK, dilution 1:6000) was used for the analysis of 7 LSCC cell lines (of the 25 collected) and total larynx tissue lysate. Furthermore, the anti-CDK1 (phospho T161) antibody (ab138389, Abcam, UK, dilution 1:750) was applied for 24 LSCC cell lines. Blots were incubated overnight in 4 °C and secondary antibody (goat anti-Rabbit, ab 97051, Abcam, UK, 1:38500) was applied (2 h, room temperature). Rabbit anti-GAPDH (ab 9485, Abcam, UK, dilution 1:2500) antibody was used as a loading control. For the protein detection, SuperSignal West Pico Chemiluminescent Substrate (Thermo Scietific, Rockford, IL USA) was used and the images were scanned and analyzed with the ChemiDoc XRS+ System (BioRad).

### Immunohistochemical analysis of laryngeal cancer formalin-fixed paraffin embedded tissue sections

The level of CDK1 protein expression was performed using automated morphometric methods in ImageJ 1.46a Program, using authors’ macro described earlier [26]. The representative microphotographs were taken at ×20 original objective magnification in the light microscope ECLIPSE E800 (Nikon Instruments Europe, Amsterdam, Netherlands), with Nikon Digital Sight DS-5Mc camera (Nikon Instruments Europe, Germany) driven by 4NIS Elements F 3.0 software (Nikon Instruments Europe, Germany). First, taken microphotographs were converted using “Colour Deconvolution” option, where the brown pigment was “isolated” from microphotography. Subsequently, using the “Threshold” option, the microphotography was converted into an 8-bit version, which allowed to creation of so-called mask. The created “mask” was applied on the original microphotography, and conversions of the color intensity into numerical data were performed. The level of CDK1 protein expression was evaluated according to modified immunoreactive scale (IRS) described first by Remmele and Stegner [27] and used (partially in modified version) in our previous publications [28–30]. The IRS was evaluated as the ratio of the percentage of positive stained cells/area (PP) and the intensity of the color reaction (SI) (IRS = SI × PP).

### Microarray DNA copy number analysis (array-CGH)

Array-CGH profiles from 13 cell lines from our previous study were used [5, 24, 25].

The log2ratio for the DNA region: chr10:62,208,242-62,223,930 covering the *CDK1* gene (according to NCBI36/hg18) Assembly) was analyzed. The mean log2 ratio of tags for the DNA region containing the *CDK1* gene was calculated. The mean log2ratio value between 0.5 and −0.5 was assumed as normal.

### The analysis of *CDK1* gene promoter DNA methylation by bisulfate pyrosequencing

DNA samples from 25 cell lines, 41 tumor samples, and controls (oral epithelium—buccal swabs, normal head, and neck tissue samples) were used. Purified DNAs were converted with bisulfite solution with the use of EpiTect DNA Modification Kit (QIAGEN, Germany), according to the manufacturer’s protocol. The primer designing and pyrosequencing protocol was performed as described elsewhere [22]. Fully methylated and unmethylated controls were used in each run. Seven CpG sequences in one CpG island were analyzed. See Supplementary data for PCR details and cutoff points calculation method. Samples with DNA methylation level above the established upper cutoff point and below the lower cutoff point were regarded as hyper- and hypomethylated, respectively.

### Sequencing analysis

Primer pairs for mutation analysis of coding exons and intron–exon junctions of *CDK1* gene were designed with the use of Primer3 v.0.4.0 online tool (http://bioinfo.ut.ee/primer3-0.4.0/). Primer details are listed in Table S3. Detailed PCR conditions are available in Supplementary data. PCR products were sequenced using Big Dye Terminator Sequencing Kit Cycle v3.1 (Applied Biosystems, Inc. (ABI), Foster City, CA, USA), according to the manufacturer’s protocol and separated using ABI PRISM 310 Genetic Analyzer (Applied Biosystems). The results were analyzed using Sequencing Analysis v. 5.2-5.4 software’s and the CodonCode Aligner software (demo mode). The reference sequence for *CDK1* gene was obtained from RefSeq database; NM_001786; UCSC Genome Browser GRCh37/hg19.

### Microarray-based microRNA expression analysis

MicroRNA expression analysis was performed using the Agilent Human microRNA Expression Microarray 66K (based on miRBase 16.0 and updates) on 16 LSCC cell lines, 5 representative primary LSCC tumor samples (out of 45 collected), and 3 non-tumor controls (Total Larynx RNA, NHBE cells and HTEC cells). To delineate the microRNAs with the highest probability to regulate *CDK1* gene, we used the following selection criteria: (1) significantly up or downregulated miRNAs, with at least twofold change as compared to controls, delineated by miRNA expression microarrays, (2) miRNAs predicted in silico to target *CDK1* by at least three miRNA databases, using MiRWalk tool (from http://www.umm.uni-heidelberg.de/apps/zmf/mirwalk/index.html) and showing at least eight seed nucleotide homology (*p* < 0.05).

## Results

### Microarray-based gene expression analysis

**Fig. 1:**
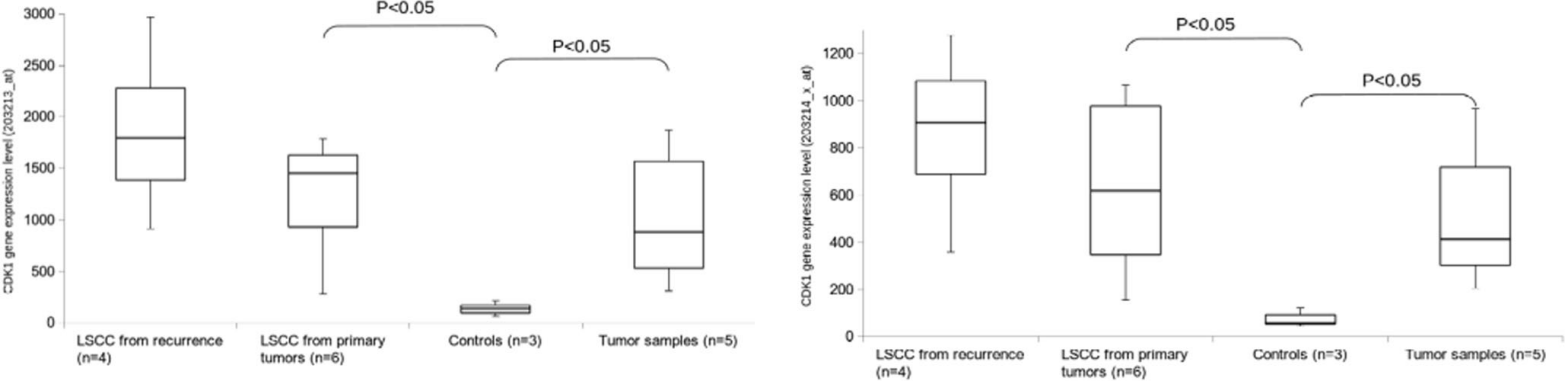
The *CDK1* gene expression analysis (microarray). *Box plots* show gene expression level in cell lines derived from primary tumors, recurrent tumors, and tumor samples in reference to non-cancer controls from head and neck region. Figure presents results obtained for two most representative tags: 203213_at (left) and 203214_x_at (right). The Mann–Whitney *U* test was performed

For all chosen genes, the expression level was higher in both cell lines and tumor samples as compared to non-cancer controls. For analyzed cyclins, the gene upregulation was shown in 9/10 (90 %) LSCC cell lines and 5/5 (100 %) tumor samples (data not shown). Among them, we choose *CDK1* for further analysis, due to its leading role in cell cycle regulation and proven oncogenic potential of this group of enzymes. For the CDK1 gene, the analyzed tags (203213_at and 203214_x_) indicated higher expression of the *CDK1* gene in all analyzed cell lines, tumor samples, and controls. The expression level was higher both in cell lines and in tumor samples as compared to non-cancer controls (*p* < 0.05; Mann-Whitney *U* test, Fig. 1). No statistically significant difference between cell lines derived from primary and recurrence tumors was observed, but the *CDK1* gene expression level was higher in primary tumor cell lines as compared to controls (*p* < 0.05, *U* test for both tags, Fig. 1). Thus, significantly higher expression level was observed in tumor samples and cell lines compared to non-cancer controls.

### Quantitative real-time PCR

**Fig. 2:**
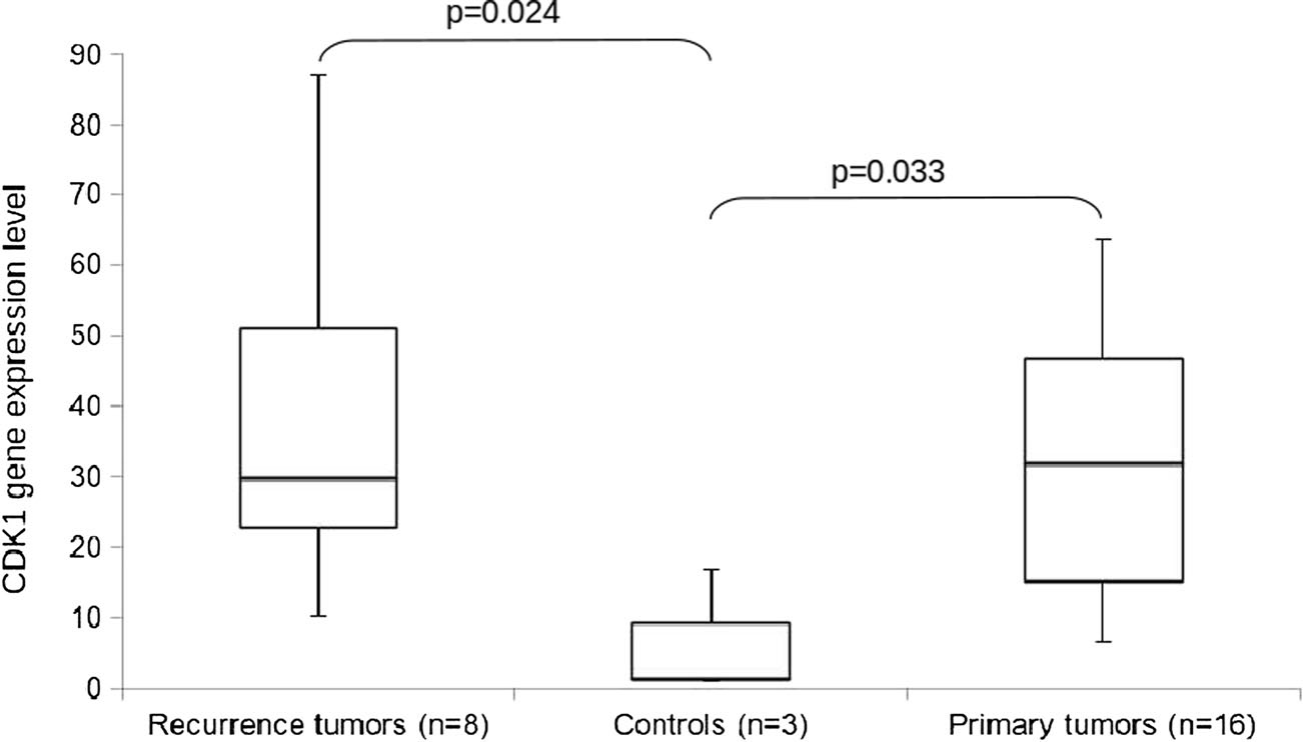
The *box plot* of RT-qPCR results showing *CDK1* gene expression levels in cell lines derived from laryngeal cancer recurrence tumors, primary tumors, and controls. The Mann–Whitney *U* test was performed. UT-SCC-6B cell line was excluded from this part of analysis as it is derived from metastasis tumor

To confirm the overexpression of the *CDK1* gene, the quantitative real-time PCR technique was performed on LSCC cell lines and non-cancer controls from head and neck region. Higher expression levels of the *CDK1* gene has been demonstrated in 9/25 (36 %) cell lines as compared to three non-cancer controls (*p* < 0.05; Mann-Whitney *U* test, Fig. 2).

Statistically significant difference between expression in LSCC cell lines in comparison to controls was observed, but there is no significant difference between cell lines derived from primary tumors and recurrent tumors (Fig. 2). The results confirmed our initial observation of the *CDK1* gene overexpression in LSCC. No correlation of *CDK1* gene expression level with tumor phenotype (TMN and grade status) or patients survival was observed.

### Western blot analysis results

**Fig. 3:**
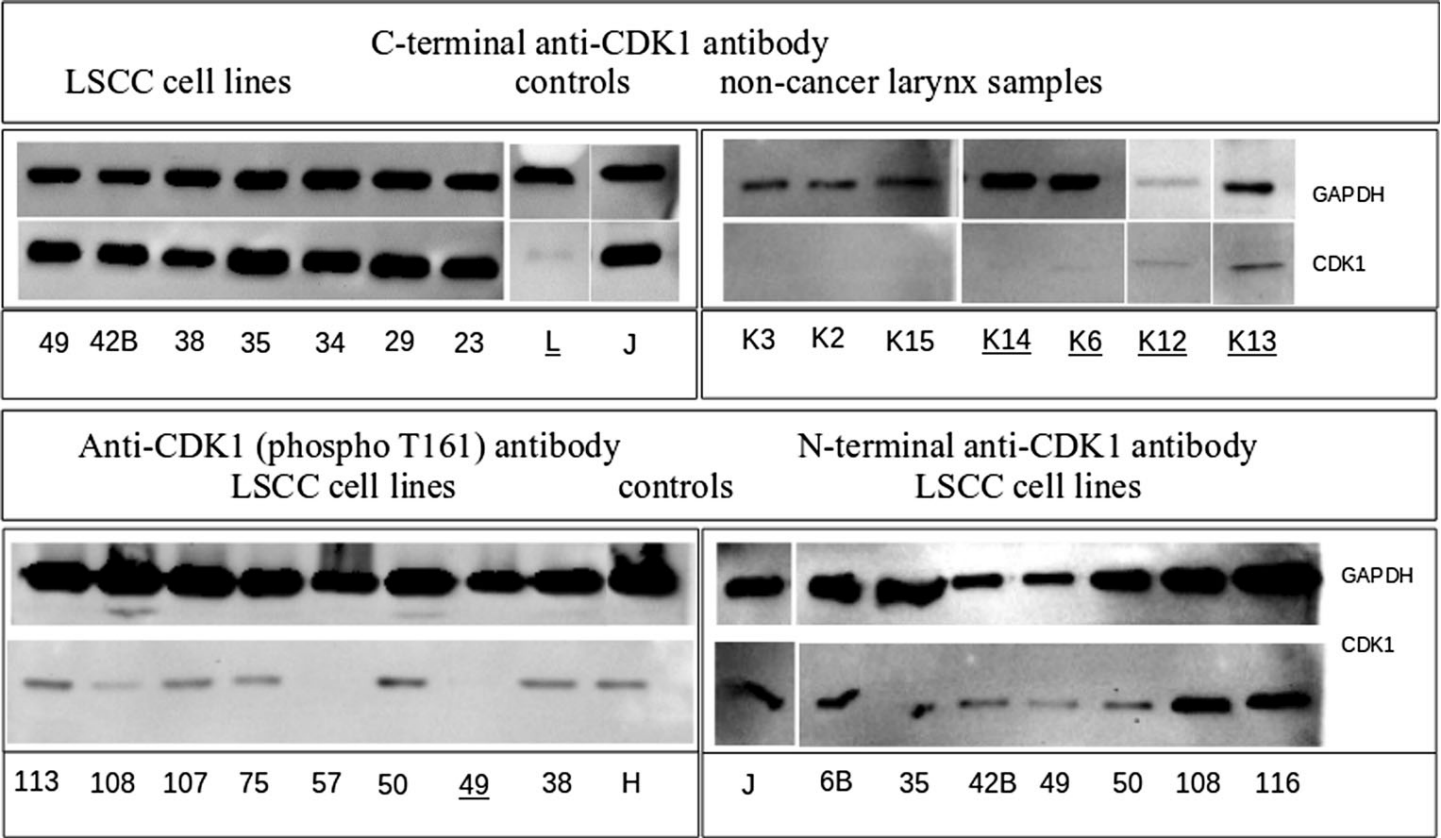
Western blot results obtained for different types of anti-CDK1 antibodies. The anti-CDK1 C-terminal antibody (*upper panel*) was applied for analysis of LSCC cell lines (*left*) and non-cancer head and neck tissue (*right*) lysates, the anti-CDK1 (phospho T161) antibody (*lower panel*, *left*) and the N-terminal anti-CDK1 antibody (*lower panel*, *right*) were applied on the LSCC cell line lysates. The *numbers* used are the LSCC numbers; *K2*, *K3*, *K6*, *K12–K15* tissue sample donors numbers, *L* total larynx tissue lysate, *J* Jurkat cell line lysate, *H* HeLa cell line lysate. GAPDH was used as a loading control and Jurkat and HeLa cell lines were the positive controls. The samples where the trace amounts of CDK1 protein were observed are *underlined*

To determine the expression of CDK1 on protein level, Western blot analysis was performed. As was shown with the C-terminal anti-CDK1 antibody, the CDK1 protein was present in all 25 analyzed cell lines. Trace amount of CDK1 protein was detected in five of the non-cancer larynx tissue samples (i.e., K6, K12, K13, and K14 and total larynx tissue lysate.) The N-terminal anti-CDK1 antibody has shown the presence of CDK1 protein in all seven LSCC cell line lysates but absent in the total larynx lysate. The analysis of 24 LSCC cell lines with the application of anti-CDK1 (phospho T161) antibody has demonstrated the presence of phosphorylated CDK1 protein in 22 LSCC cell lines; in 1 LSCC cell line (UT-SCC-49) trace amount of analyzed protein was observed; and in one LSCC cell line (UT-SCC-57), lack of phosphorylated CDK1 protein was shown. The positive control lysates has demonstrated the presence of CDK1 protein, and the loading control—GAPDH protein was present in all analyzed tissue lysates. For all the analyzed proteins, one band with adequate size was observed (Fig. 3).

This analysis has shown the presence of the CDK1 protein in LSCC and its absence or small amounts in controls derived from non-cancer tissue from the larynx or regions surrounding the larynx. As was shown, in LSCC, the CDK1 is present in active-phosphorylated form. Moreover, this analysis shown the lack of the changes in protein length resulting from the CDK1 gene polymorphism rs3212319.

### IHC analysis results

**Fig. 4:**
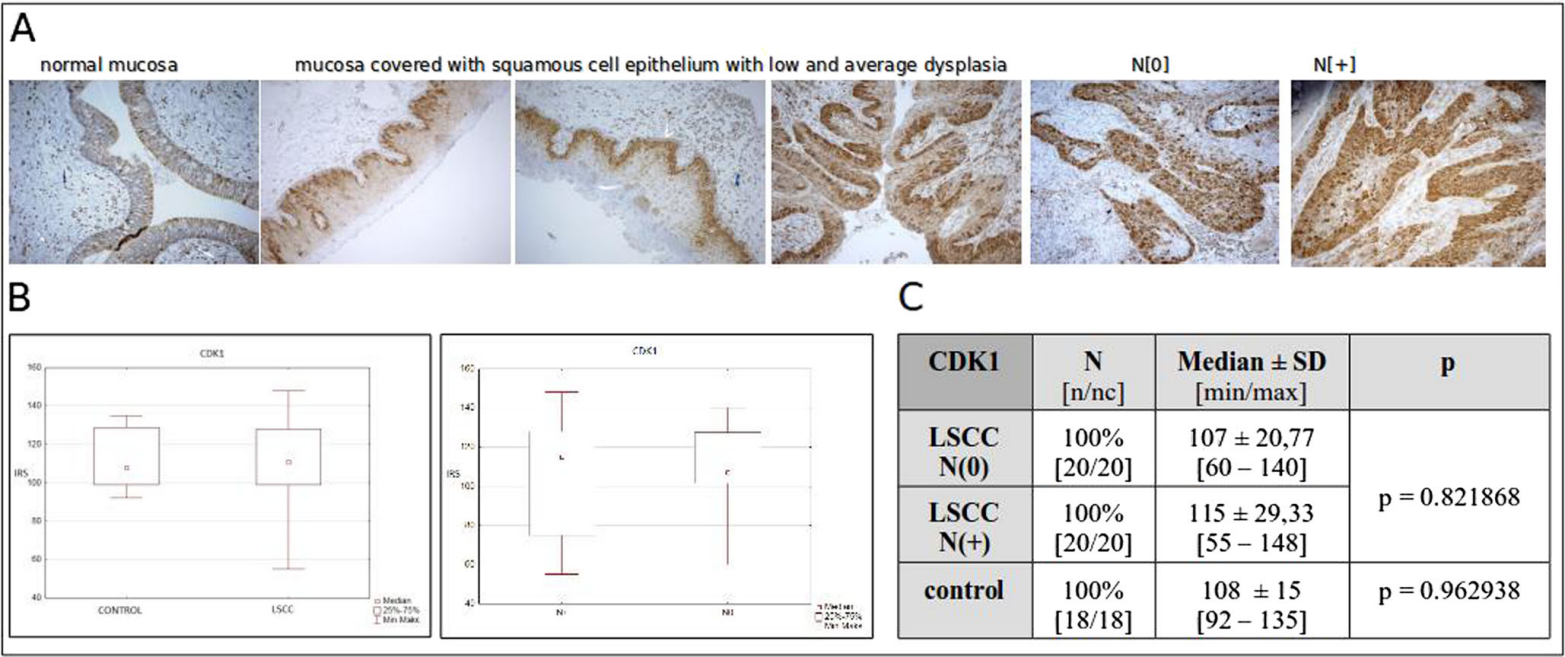
Immunohistochemical representative microphotographs representing the cdk-1 expression in normal mucosa (represented as columnar epithelium); on the following three micrographs, there are presented fragments of mucosa covered with squamous cell epithelium with low and average dysplasia, then primary LSCC in patients without lymph node metastases N (0), in LSCC in patients with lymph node metastases N (+). *Brown color* corresponds to localization of CDK1 antigen, antibody complex visualized by peroxidase system, and developed by DAB as a chromogen, nucleus counterstained with hematoxylin. Primary objective magnification ×10

The immunohistochemical studies were performed to evaluate the localization of CDK1 protein expression in LSCC primary tumors, as well as for description of the differences in protein expression (cytoplasmic vs. nuclear) in research LSCC group of patients. The analyses showed nuclear-cytoplasmic expression of CDK1 protein in all 40/40 (100 %) primary LSCC and in all 18/18 (100 %) cases of normal mucosa (Fig. 4a), but higher CDK1 expression in LSCC tumors median [CDK1 IRS = 111.00] compared to normal mucosa median [CDK1 IRS = 108.00] was observed. In 20/40 LSCC cases, we have revealed the nuclear expression of CDK1 protein; the remaining 20/40 cases revealed only cytoplasmic expression of analyzed antigen. No significant differences in expression of the studied protein was observed between primary LSCC and control group (*p* = 0.962938) (Fig. 4b,c).

According to lymph node involvement, we have revealed lower CDK1 protein expression in patients without lymph node metastases (CDK1IRS N (0) = 107) compared to the patients with lymph node involvement (CDK1IRS N (+) = 115) (Fig.4b,c). Moreover, in LSCC N (0) group, 9/20 cases revealed nuclear expression of CDK1, and in LSCC N (+) group, 11/20 cases revealed the nuclear expression of established antigen. No significant differences in expression of the studied protein was observed in the analyzed groups (*p* = 0.821868). Whereas, no significant differences between nuclear and cytoplasmic expression, as well as lack of correlation between nuclear-cytoplasmic ratio of CDK1 expression according lymph node metastases was shown. The differentiated pattern of CDK1 expression profile may indicate the heterogeneity of the LSCC.

### Microarray-based DNA copy number analysis

We first analyzed available array-CGH profiles to check for possible copy number gains of the *CDK1* locus in the 13 LSCC cell lines. In all instances, the mean log2 ratio for analyzed LSCC cell lines ranged from −0.20 to 0.15. Therefore, in all 13 cell lines, DNA copy number changes in the *CDK1* locus cannot account for the observed evaluated expression level of the *CDK1* gene in LSCC.

### *CDK1* gene sequencing results

As no copy number alterations, that could explain elevated expression of *CDK1*, were identified, we screened for potential activating mutations in coding sequences of the gene. Two common SNP variants were found in the analyzed samples: rs3212319 (15/25 cell lines) and rs1871446 (24/25 cell lines). The rs3212319 polymorphism is an intronic variant (NC_000010.10: g.62551816delC; NM_001786.4: c.653 + 5delC; (GRCh37/hg19)) and results in a deletion of the cytosine in the position +5 in intron 6. Of the 15 affected samples, 13 were heterozygotes (C/-) and 2 were deleted in a homozygous manner (−/−). The rs1871446 polymorphism is a 3′UTR variant and results in a A > G substitution (NC_000010.10: g.62553763A > G; NM_001786.4: c.*30A > G; UCSC (GRCh37/hg19). Of the 24 affected samples, 4 were heterozygous alterations (A/G), while the rest were homozygotes (G/G). No other alterations in coding sequence or exon-intron junction site were detected. Therefore, regarding the expression data, most probably none of these SNP is associated with the elevated *CDK1* observed in LSCC.

### DNA methylation analysis of *CDK1* promoter region by pyrosequencing

The changes in pattern of DNA methylation is one of the mechanisms of the gene transcription regulation. DNA methylation level of *CDK1* gene promoter was analyzed in all cell lines and primary tumor samples. The buccal swabs and non-cancer head and neck tissue samples were used as controls. The normal DNA methylation level was estimated to range between 1.59 and 13.7 %. The mean methylation for the LSCC cell lines ranged between 3.33 and 6.81 % and for the tumor samples ranged 0 %–1.46 %. Therefore, no meaningful differences were found between the samples. Thus, the *CDK1* gene hypomethylation was excluded as a mechanism of the gene upregulation.

### Microarray-based microRNA expression analysis

Lastly, we analyzed another epigenetic mechanism that on account for changes in gene expression—miRNAs. We delineated the group of microRNAs with significant downregulated expression between 16 cell lines, 5 primary tumor samples, and 3 controls. The analysis has revealed 12 downregulated miRNA genes. None of this was reported to be involved in *CDK1* gene regulation. Therefore, we failed to delineate any miRNA which could regulate *CDK1* expression level in the analyzed cases.

## Discussion

In the current study, we have analyzed the expression pattern of group of genes —*CCNB1*, *CCNB2*, *CCNA2*, and *CDK1* in laryngeal squamous cell carcinoma. All of the chosen genes are important factors in eukaryotic cell cycle. We have shown that all the analyzed genes are upregulated in LSCC cell lines and tumor samples as compared to non-cancer head and neck controls. From this group of genes, we have chosen the *CDK1* gene for further analysis in context of searching the new potential oncogenes in LSCC. The main criterion we assumed for choosing this gene was its role in cell cycle control. *CDK1* is a member of the Ser/Thr protein kinase family, and its product is a catalytic subunit of M-phase promoting factor, essential for G1/S and G2/M phase transitions of eukaryotic cell cycle. The oncogenic character of Ser/Thr protein kinases like B-Raf and PIM1 was shown [12–14]. It was demonstrated that *CDK1* is suifficient to drive the cell cycle in mammalian cells [31] which indicates the leading role of this kinase in cell cycle regulation. Moreover, as was shown in previous papers, *CDK1* gene phosphorylates a group of transcription factors (TF) involved in cell cycle control and cell proliferation [32, 33].

Nowadays, large field of cancer studies focuses on the identification of novel, potential oncogenes or tumor suppressor genes. The detailed knowledge of tumor genetic background is fundamental for establishing new diagnostic and treatment strategies, including targeted therapy. Genetic data have been successfully transformed into clinical practice, for example for chronic myeloid leukemia (CML) where the application of BCR-ABL tyrosine kinase inhibitor—imatinib is widely used [34], and in case of HER2-positive breast cancer, combination of lapatynib and transtuzumab is applied for inhibition of HER2 [35]. So far, for head and neck tumors, only Cetuximab has been approved for treatment [36]. Here, we have analyzed a potential target for an inhibition therapy—*CDK1* in larynx squamous cell carcinoma (LSCC) and show its overexpression in this tumor. Importantly, Goga et al. showed that the inhibtion of *CDK1* gene leads to downregulation of survivin expression and induction of MYC-dependent apoptosis [37]. Moreover, the *CDK1* gene silencing with the application of RNAi was indicated as a novel potential tool for the therapy of malignant pleural mesothelioma [38], epithelial ovarian cancer [39], and breast cancer [40, 41]. The in-depth analysis of our microarray expression profiles showed higher *CDK1* expression in both larynx cancer cell lines and primary tumor samples in comparison to normal controls. This observation was further confirmed with the application of quantitative real-time PCR technique. This is in line with previous results showed by Lian et al. in larynx cancer tissues compared to adjacent non-neoplastic tissues [17]. Besides, *CDK1* overexpression in cancer was shown also for oral squamous cell carcinoma [42], breast cancer [43], epithelial ovarian cancer [39], and hepatocellular carcinoma [44].

To confirm overexpression of *CDK1* on protein level, we performed the Western blot analysis. Our results indicated that the expression of CDK1 protein is absent or very weak in most of the normal larynx tissue as compared to LSCC cell lines. Moreover, in LSCC cell lines, the CDK1 protein was characteristic for phosphorylation at threonine 161, and it was shown that this is a feature of CDK1 activation [45]. However, the immunohistochemical analysis of laryngeal cancer tissue sections has revealed that the CDK1 protein is present both in cancer and non-cancer tissue samples (histopathologically normal surgical margins of laryngeal tumors). We suppose that these inconsistencies result from the differences of routinely used control samples. For the Western blot analysis, the control samples consisted of commercially offered total larynx tissue lysates and samples derived from the non-cancer disease interventions in the vicinity of the larynx, and as such, material is free from cancerogenic process. In samples derived from the other non-cancer samples, the trace amounts of CDK1 protein were observed. On the other hand, the control samples applied in immunohistochemistry (IHC) studies were derived from surgical margins of laryngeal tumors. The literature data indicate that such material, even if histopathologically normal, may contain genetic alterations appearing as the result of field cancerization process [46]. This is exemplified by the fact that second primary tumors frequently develop in patients after laryngectomy. Thus, the surgery margins contain cells that can transform into cancer. It is probable then that deregulation of *CDK1* gene expression and the altered protein level occurs on very early step of carcinogenesis. The role of CDK1 protein overexpression in surgery margins of laryngeal cancer was noticed by Yang and coworkers [16] who had shown its relation with the local relapse occurrence. The CDK1 protein overexpression in OSCC was also shown in Xin Chen group, who had shown the presence of CDK1 protein in non-cancer epithelium in 35 % of OSCC cases and in 67.5 % of oral cancer samples [47].

We have also analyzed the nuclear-cytoplasmic ratio of CDK1 expression. In our study, no significant changes were found in the LSCC group as compared to normal mucosa samples. Also, no differences were shown within the LSCC samples group when the N0 group was compared to N+ group. This is in contrast to findings from colorectal tumor when it has been shown that high nuclear/cytoplasmic ratio of CDK1 expression is connected with poor prognosis in this type of cancer [48].

We further seek to identify mechanisms that potentially underlay the altered expression of *CDK1* gene. We searched for activated mutation by Sanger sequencing that could possible explain *CDK1* overexpression. In this analysis, two nucleotide polymorphisms (SNP)—rs1871446 and rs3212319 were detected. In our study, in 25 LSCC cell lines for polymorphism rs1871446, 1 homozygous (A/A), 4 heterozygous alterations (A/G), and 20 homozygotes (G/G) were detected. No correlation of analyzed SNP with patient survival time was observed; thus, we did not confirm any influence of GG homozygotes on the course of laryngeal cancer. Interestingly the second polymorphism, rs3212319 results in a deletion of cytosine in +5 position of intron 6. Of 15 affected samples, 13 were heterozygotes (C/−), and 2 were deleted in a homozygous manner (−/−). To verify whether this sequence variation induces changes in CDK1 protein length, the N-terminal anti-CDK1 antibody was used for the Western blot approached. Lysates of seven LSCC cell lines (two heterozygotes and two altered homozygotes) were used. The results of this analysis suggest that rs3212319 has no influence on CDK1 protein length. Moreover, no correlations of the observed polymorphisms with expression level of *CDK1* gene on both mRNA and protein level in LSCC cell lines were found.

Therefore, using array-CGH, we excluded that gain of copy number of the *CDK1* gene is responsible for the transcriptional upregulation. Further, we found no differences in the gene promoter methylation level between in LSCC cell lines, primary tumors, and control samples. Therefore, we conclude that lack of DNA methylation in *CDK1* promoter region comprises a normal condition found in healthy controls and thus cannot be considered as the mechanism driving *CDK1* overexpression.

Lastly, we hypothesized that *CDK1* overexpression may be connected with downregulation of a microRNA that regulate the gene. It is already known that microRNA-7 and miR-490-3-p target *CDK1* gene [49, 50]. Interestingly, microRNA-7 is downregulated in adrenocortical carcinoma and transfected to the cells and leads to the inhibition of *CDK1* gene. Moreover, a group of other microRNAs was identified as potentially involved in the regulation of *CDK1* gene in laryngeal cancer. Among them, the hsa-miR-139, hsa-miR-203, and miR-145 downregulation were observed in larynx and hypopharynx tumor samples [51, 52]. However, our study did not confirm these data—no changes in the expression profile of any of these microRNAs were observed using a microarray profiles.

## Conclusion

In summary, our results show that *CDK1* gene expression is recurrently elevated in LSCC. Moreover, it suggests that the alterations may appear in the very early step of carcinogenesis, as its mRNA and protein abundance is observed in surgery margin and T1 tumors. These data and the important role of CDK1 in cell cycle regulation and control as well as the proven impact of gene silencing on the tumor cell growth imply that this gene may function as a potential oncogene. However, the confirmation of the oncogenic character of *CDK1* gene requires further study. Our attempts to identify the molecular mechanisms failed because we did not observe significant changes neither in the DNA sequence nor in the gene copy number. Similarly, the pyrosequencing and miRNA expression analyses did not reveal any differences in methylation level and miRNA expression, respectively; thus these mechanisms probably do not contribute to elevated of *CDK1* expression observed in LSCC.

## Electronic supplementary material

Below is the link to the electronic supplementary material.

ESM 1 (DOCX 18 kb)

ESM 2 (DOCX 14 kb)

ESM 3 (DOCX 15 kb)

ESM 4 (DOCX 15 kb)
