# Supplementary material for: Recurrent CDK1 overexpression in laryngeal squamous cell carcinoma
Source: Tumour Biol. 2016 Feb 24;37(8):11115–26. doi: 10.1007/s13277-016-4991-4 (PMC4999469; doi:10.1007/s13277-016-4991-4)
Supplement: Supplementary file 1 — (DOCX 18 kb) [file 13277_2016_4991_MOESM1_ESM.docx]

*CDK1* as potential oncogene in laryngeal squamous cell carcinoma

Tumor biology

Bednarek K.^1^, Kiwerska K.^1^, Szaumkessel M.^1^, Bodnar M.^2^, Kostrzewska-Poczekaj M.^1^, Marszałek A.^2,3^, Janiszewska J.^1^, Bartochowska A.^4^, Jackowska J.^4^, Wierzbicka M.^4^, Grenman R.^5^, Szyfter K.^6^, Giefing M.^1,4^, Jarmuż-Szymczak M^1,7^.

1. Institute of Human Genetics, PAS, Department of Cancer Genetics, Poznan, Poland

# Department of Clinical Pathomorphology, Collegium Medicum, Nicolaus Copernicus University, Bydgoszcz, Poland

# Department of Oncologic Pathology, Greater Poland Cancer Centre, Poznan, Poland

1. Department of Otolaryngology and Laryngological Oncology, University of Medical Sciences, Poznan, Poland
2. Department of Otorhinolaryngology - Head and Neck Surgery and Department of Medical Biochemistry, Turku University Hospital and University of Turku, Turku, Finland

# Department of Audiology and Phoniatry, University of Medical Sciences, Poznan, Poland

# Department of Hematology, University of Medical Sciences, Poznan, Poland

e-mail:maljar@man.poznan.pl

Supplemental data

**Reverse transcription and quantitative real-time PCR The PCR reaction was started with the incubation at 95ºC for 15 minutes for polymerase activation and next cycled 40 times with following conditions: denaturation - 95°C for 20 sec, annealing - 55°C for 10 sec, elongation - 72°C for 20 sec. The annealing temperature applied for all genes was 55°C.**

**CDK1 protein Western Blot analysis**

For protein analysis, after the denaturation in 4xLaemmli sample buffer (Laemmli 1970) in 95°C for 5 minutes the SDS-PAGE electrophoresis in 4-12% polyacrylamide gel with TRIS-glicyne running buffer (25 mM Tris base, 190 mM glycine) was performed (Mini protean, Bio-Rad). Twenty micrograms of total protein per lane was loaded. Next, the wet transfer on PVDF membrane was performed (transfer buffer: 25 mM Tris base, 190 mM glycine,10% methanol; 1 hour, 100V, 4°C). PBS-T (25 mM Tris, 150 mM NaCl, 2 mM KCl, pH 7.4 with 0.05% Tween-20) with 5% non-fat dry milk was used as a blocking reagent and antibodies diluent. Blots were blocked for 1 hour in 4°C and next incubated with one of three types of anti-CDK1 primary antibodies.

**Immunohistochemical analysis of laryngeal cancer formalin fixed paraffin embedded tissue sections**

Statistical analysis was performed using STATISTICA 10 (StatSoft, Polish Version, Poland). The normal distribution was tested using the Kolmogorov–Smirnov test with Lilliefors correction and the Shapiro–Wilk test. The variance was assessed using Levene's test. For the statistical analysis nonparametric U Mann–Whitney test was used, and p < 0.05 was considered as statistically significant difference.

***CDK1* gene promoter region DNA methylation analysis bisulfate - Pyrosequencig**

For the pyrosequencing the PCR primers sequences (5’-3’) were as follows: Forward: TGGTTTTAAAGTTGGTTTTTGGAAA, Reverse: ACCCTAACCCCAACCACTATA and Sequencing: TGGTTTTTGGAAATTGAG. The PCR reaction was performed according to protocol supplied by PyroMark PCR kit (Qiagen, Germany) with annealing temperature 60°C. The PCR product length was 110 bp. The normal DNA methylation level range was defined by two values of cut-off. The upper cut-off point was calculated according to the scheme: the mean DNA methylation for the oral epithelium and head and neck tissues plus 3 times standard deviation for DNA samples from the controls. The lower cut-off point was the lowest value of mean DNA methylation for the control samples.

**Sequencing analysis**

The PCR amplification was performed with BioRad DNA Engine Dyad Peltier Thermal Cycler .The amplification was carried with in total volume 10µl containing 50ng of DNA template and 10× buffer: 100 mM Tris-HCl, pH 8.3 at 25°C; 500 mM KCl; 15 mM MgCl_2_; 0.01% gelatin (Sigma Aldrich), 0.2 µM each dNTPs (Roche Diagnostic, Germany), 0.2 µM each primers, 0.8µl MgCl_2_ (25mM conc., Fermentas). The PCR amplification was performed with the use of Taq Polymerase (Sigma or Fermentas). The PCR conditions was 95°C for 10 minutes and next 34 times cycles as follows: denaturation 95°C for 15 sec, annealing 60°C for 15 sec, elongation 72°C for 40 sec finished by final elongation 72°C for 10 minutes. PCR products were obtained with application of Taq DNA polymerase (Fermentas or Sigma), analyzed in agarose gel stained with ethidium bromide and then were purified with ExoSAP-IT reagent (USB Corporation, Cleveland, OH, USA).
