# Supplementary material for: Recurrent CDK1 overexpression in laryngeal squamous cell carcinoma
Source: Tumour Biol. 2016 Feb 24;37(8):11115–26. doi: 10.1007/s13277-016-4991-4 (PMC4999469; doi:10.1007/s13277-016-4991-4)
Supplement: Supplementary file 2 — (DOCX 14 kb) [file 13277_2016_4991_MOESM2_ESM.docx]

*CDK1* as potential oncogene in laryngeal squamous cell carcinoma

Tumor biology

Bednarek K.^1^, Kiwerska K.^1^, Szaumkessel M.^1^, Bodnar M.^2^, Kostrzewska-Poczekaj M.^1^, Marszałek A.^2,3^, Janiszewska J.^1^, Bartochowska A.^4^, Jackowska J.^4^, Wierzbicka M.^4^, Grenman R.^5^, Szyfter K.^6^, Giefing M.^1,4^, Jarmuż-Szymczak M^1,7^.

1. Institute of Human Genetics, PAS, Department of Cancer Genetics, Poznan, Poland

# Department of Clinical Pathomorphology, Collegium Medicum, Nicolaus Copernicus University, Bydgoszcz, Poland

# Department of Oncologic Pathology, Greater Poland Cancer Centre, Poznan, Poland

1. Department of Otolaryngology and Laryngological Oncology, University of Medical Sciences, Poznan, Poland
2. Department of Otorhinolaryngology - Head and Neck Surgery and Department of Medical Biochemistry, Turku University Hospital and University of Turku, Turku, Finland

# Department of Audiology and Phoniatry, University of Medical Sciences, Poznan, Poland

# Department of Hematology, University of Medical Sciences, Poznan, Poland

e-mail:maljar@man.poznan.pl

Table S1. The TNM and G status details for primary tumor samples used for the gene and microRNA expression analysis, pyrosequencing and immunohistochemistry.

| Primary tumor samples used for the gene and microRNA expression analysis and pyrosequencing | | Primary tumor samples used for the immunohistochemistry | |
| --- | --- | --- | --- |
| TNM status | number of samples | TNM status | number of samples |
| T1 | 7 | pT2 | 1 |
| T3 | 18 | pT3 | 27 |
| T4 | 20 | pT4 | 12 |
| N0 | 21 | pN0 | 20 |
| N(+) | 24 | pN(+) | 20 |
| M0 | 44 | M0 | 40 |
| M1 | 1 | M(+) | 0 |
| G1 | 18 | G1 | 2 |
| G2 | 20 | G2 | 28 |
| G3 | 7 | G3 | 1 |
|  |  | Gx | 9 |
