# Supplementary material for: Recurrent CDK1 overexpression in laryngeal squamous cell carcinoma
Source: Tumour Biol. 2016 Feb 24;37(8):11115–26. doi: 10.1007/s13277-016-4991-4 (PMC4999469; doi:10.1007/s13277-016-4991-4)
Supplement: Supplementary file 3 — (DOCX 15 kb) [file 13277_2016_4991_MOESM3_ESM.docx]

*CDK1* as potential oncogene in laryngeal squamous cell carcinoma

Tumor biology

Bednarek K.^1^, Kiwerska K.^1^, Szaumkessel M.^1^, Bodnar M.^2^, Kostrzewska-Poczekaj M.^1^, Marszałek A.^2,3^, Janiszewska J.^1^, Bartochowska A.^4^, Jackowska J.^4^, Wierzbicka M.^4^, Grenman R.^5^, Szyfter K.^6^, Giefing M.^1,4^, Jarmuż-Szymczak M^1,7^.

1. Institute of Human Genetics, PAS, Department of Cancer Genetics, Poznan, Poland

# Department of Clinical Pathomorphology, Collegium Medicum, Nicolaus Copernicus University, Bydgoszcz, Poland

# Department of Oncologic Pathology, Greater Poland Cancer Centre, Poznan, Poland

1. Department of Otolaryngology and Laryngological Oncology, University of Medical Sciences, Poznan, Poland
2. Department of Otorhinolaryngology - Head and Neck Surgery and Department of Medical Biochemistry, Turku University Hospital and University of Turku, Turku, Finland

# Department of Audiology and Phoniatry, University of Medical Sciences, Poznan, Poland

# Department of Hematology, University of Medical Sciences, Poznan, Poland

e-mail:maljar@man.poznan.pl

Tab S2. PCR primers sequences and amplification conditions for RT-QPCR. Letter “i” after the amplicon length indicates that the exon/exon boundary was inside the amplified sequence.

| **Gene name**  NCBI Reference Sequence | Primers sequences | Amplicon length (bp) | Annealing Tm (°C) | PCR Efficiency |
| --- | --- | --- | --- | --- |
| ***CDK1*** NM_001170406 | \| F: 5’ CAGACTAGAAAGTGAAGAGGAAGG 3’ \| \| --- \| \| R: 5’ ACTGACCAGGAGGGATAGAATC 3’ \| | 191-i | 55 | 100% |
| ***UBC***  NM_021009 | \| F: 5’ TCGCAGTTCTTGTTTGTG 3’ \| \| --- \| \| R: 5’ GATGCCTTCCTTATCTTGG 3’ \| | 150-i | 55 | 100% |
| ***ARNT***  NM_001668 | \| F: 5’ TTGGCAGCACACTCTATG 3’ \| \| --- \| \| R: 5’ C--CTCATTCGGCAAATAAACG 3’ \| | 191-i | 55 | 100% |
| ***GAPDH*** NM_002046 | \| F: 5’ CGGAGTCAACGGATTTGGTCGTAT 3’ \| \| --- \| \| R: 5’ AGCCTTCTCCATGGTGGTGAAGAC 3’ \| | 307-i | 55 | 99% |
