# Supplementary material for: Recurrent CDK1 overexpression in laryngeal squamous cell carcinoma
Source: Tumour Biol. 2016 Feb 24;37(8):11115–26. doi: 10.1007/s13277-016-4991-4 (PMC4999469; doi:10.1007/s13277-016-4991-4)
Supplement: Supplementary file 4 — (DOCX 15 kb) [file 13277_2016_4991_MOESM4_ESM.docx]

*CDK1* as potential oncogene in laryngeal squamous cell carcinoma

Tumor biology

Bednarek K.^1^, Kiwerska K.^1^, Szaumkessel M.^1^, Bodnar M.^2^, Kostrzewska-Poczekaj M.^1^, Marszałek A.^2,3^, Janiszewska J.^1^, Bartochowska A.^4^, Jackowska J.^4^, Wierzbicka M.^4^, Grenman R.^5^, Szyfter K.^6^, Giefing M.^1,4^, Jarmuż-Szymczak M^1,7^.

1. Institute of Human Genetics, PAS, Department of Cancer Genetics, Poznan, Poland

# Department of Clinical Pathomorphology, Collegium Medicum, Nicolaus Copernicus University, Bydgoszcz, Poland

# Department of Oncologic Pathology, Greater Poland Cancer Centre, Poznan, Poland

1. Department of Otolaryngology and Laryngological Oncology, University of Medical Sciences, Poznan, Poland
2. Department of Otorhinolaryngology - Head and Neck Surgery and Department of Medical Biochemistry, Turku University Hospital and University of Turku, Turku, Finland

# Department of Audiology and Phoniatry, University of Medical Sciences, Poznan, Poland

# Department of Hematology, University of Medical Sciences, Poznan, Poland

e-mail:maljar@man.poznan.pl

Tab. S3. Primers sequences and amplicon lengths of *CDK1* gene

| *CDK1* gene exon number | Primers sequences (5’-3’) | Annealing temperature (°C) | Amplicon size (bp) |
| --- | --- | --- | --- |
| Exon 1 | Exon in non-coding region |  |  |
| Exon 2 | F: CACGTTTCCAATGTCTCAGG  R: CGGTCATTAGGGATTCGGTA | 60 | 478 |
| Exon 3 | F: CAAGACCCTGCCATAAGGAA  R: TGTGCGGCATTCTCAACTAC | 60 | 396 |
| Exon 4 | F: GTTGCCCTGAGATTCCTTTC  R: CCACAAAATGCAGGGACTTC | 60 | 380 |
| Exon 5 | F: GCCTAAAATGGCCTGAAAGC  R: CTCCTGCCATGTCCCTCTAG | 60 | 545 |
| Exon 6+7 | F: TTGGTGGCAGTCATACAACC  R: TTTTCTAGGCAAAACAAAGAACTG | 60 | 591 |
| Exon 8 | F: TGAAAGTATTAGTTTTGGTTTATTGC  R: CGAAGTACAGCTGAAGTTTGATAAC | 60 | 326 |
